# Impact of severe postoperative complications on the prognosis of older patients with colorectal cancer: a two-center retrospective study

**DOI:** 10.1186/s12876-024-03213-y

**Published:** 2024-04-02

**Authors:** Daiki Matsubara, Koji Soga, Jun Ikeda, Tatsuya Kumano, Masato Mitsuda, Tomoki Konishi, Shuhei Komatsu, Katsumi Shimomura, Fumihiro Taniguchi, Yasuhiro Shioaki, Eigo Otsuji

**Affiliations:** 1 Department of Gastroenterological Surgery, Japanese Red Cross Kyoto Daiichi Hospital, 15-749, Honmachi, Higashiyama-ku, 605-0981 Kyoto, Japan; 2 Department of Surgery, Japanese Red Cross Maizuru Hospital, 427, Kuratani, Maizuru, 624-0906 Kyoto, Japan; 3 Department of Surgery, National Hospital Organization Maizuru Medical Center, 2410, 625-0052 Yukinaga, Maizuru, Kyoto, Japan; 4 Division of Digestive Surgery, Department of Surgery, Kyoto Prefectural University of Medicine, 465 Kajii-cho, Kawaramachihirokoji, Kamigyo-ku, 602-0841 Kyoto, Japan

**Keywords:** Colorectal cancer, Older patients, Severe postoperative complication, Minimally invasive surgery

## Abstract

**Background:**

The occurrence of postoperative complications may affect short-term outcomes and prognosis of patients with various malignancies. However, the prognostic impact of these complications in older patients with colorectal cancer (CRC) remains unclear. Therefore, this study aimed to investigate the impact of severe postoperative complications on the oncological outcomes of older (aged ≥ 80 years) and non-older (aged < 80 years) patients with CRC.

**Methods:**

We retrospectively analyzed 760 patients with stage I–III CRC who underwent curative surgery in two institutions between 2013 and 2019. The patients were categorized into older (aged ≥ 80 years, 191 patients) and non-older (aged < 80 years, 569 patients) groups. Short- and long-term outcomes were compared between the two groups.

**Results:**

The incidence of severe postoperative complications did not differ between the two groups (*p* = 0.981). Cancer-specific survival (CSS) was significantly worse in older patients with severe complications than in those without severe complications (*p* = 0.007); meanwhile, CSS did not differ between the non-older patients with severe complications and those without severe complications. Survival analysis revealed that the occurrence of severe postoperative complications was an independent prognostic factor for CSS in older patients (hazard ratio = 4.00, 95% confidence interval: 1.27–12.6, *p* = 0.017).

**Conclusion:**

CRC surgery can be safely performed in older and non-older patients. Moreover, the occurrence of severe postoperative complications might more strongly affect the prognosis of older patients than that of non-older patients.

**Supplementary Information:**

The online version contains supplementary material available at 10.1186/s12876-024-03213-y.

## Background

Colorectal cancer (CRC) had the third highest incidence rate (over 900,000 new cases globally) and the second highest cancer-related mortality rate in 2020 [1]. Prolonged life expectancy has led to the increasing incidence of CRC in older patients, with 25% of individuals with CRC being diagnosed at the age of ≥ 80 years [2]. Owing to the increased number of older patients who undergo surgery for CRC and the prolonged life expectancy of older individuals, the prognostic predictors of CRC in older patients should be elucidated.

Generally, older patients have more comorbidities that may affect their postoperative course. The occurrence of postoperative complications can impair the quality of life and be associated with mortality. Therefore, postoperative morbidity and mortality rates are higher in older patients [3, 4]. Nevertheless, recent studies have demonstrated the technical and oncological safety of laparoscopic surgery in older patients [5, 6]. However, the occurrence of postoperative complications could affect not only the short-term outcomes but also the prognosis in patients with various malignancies [7–9]. The prognostic impact of these complications in older patients with CRC remains unclear.

Therefore, this study aimed to evaluate the short- and long-term outcomes in older patients with CRC. Moreover, whether the postoperative complications affect the prognosis in patients aged ≥ 80 years with CRC was investigated using the cancer-specific survival (CSS) rate, and the results were compared with those in non-older patients.

## Methods

### Patients

We retrospectively analyzed 763 patients with pathological stage (pStage) I–III CRC who underwent curative surgery in two institutions between 2013 and 2019. Three patients, aged 77, 85, and 88 years, who died during their hospital stay were excluded. Hence, only 760 patients were included in this study. Of the total patients, 660 and 100 were treated in Hospitals 1 and 2, respectively. The patients were categorized into the older (aged ≥ 80 years, *n* = 191) and non-older (aged < 80 years, *n* = 569) groups. Their characteristics, pathological and surgical findings, and postoperative clinical courses were reviewed retrospectively from the medical records and databases of our institutions. The preoperative diagnosis of colorectal adenocarcinoma was confirmed through endoscopy, followed by biopsy. Colorectomy and lymph node dissection were performed in accordance with the Japanese Society for Cancer of the Colon and Rectum [10]. Tumor staging was performed based on the 8th Union for International Cancer Control staging system [11]. The macroscopic and histological types were determined according to the Japanese classification of colorectal carcinoma, 9th edition [12]. Follow-up procedures were performed every 3–6 months for 2 years postoperatively and continued for at least 5 years.

### Evaluation of postoperative complication

The presence and severity of postoperative complications were evaluated using the Clavien–Dindo classification; severe complications were defined as grade III or higher overall complications [13, 14].

### Statistical analyses

The continuous variables were expressed as the mean ± standard deviation. The categorical variables between the two groups were compared using the chi-square test. The survival curves for overall survival (OS), CSS, and estimated cumulative incidence of recurrence were derived using the Kaplan–Meier method and compared using the log-rank test. CSS was defined as the time between the date of surgery and date of death from CRC. To analyze the impact of complications on prognosis of CRC and minimize the effect of early deaths that resulted from postoperative complications, patients who died during their hospital stay were excluded from survival analysis. Multivariate survival analysis was performed using the Cox proportional hazard regression model to identify the independent prognostic factors for CSS. The propensity score of each patient was assessed using a logistic regression model to control confounding factors for the occurrence of severe postoperative complications and prognosis. The covariates included sex (male or female), body mass index (> 22 or ≤ 22 kg/m^2^), American Society of Anesthesiologists (ASA) Physical Status (1–2 or ≥ 3), tumor location (right-/left-sided colon/rectum), treatment for obstruction (present or absent), histopathological type (tub and pap/por and muc), pathological T stage (T1-3 or T4) and N status (N0 or N1-2), stoma (permanent/diverting/absent), surgical approach (open or laparoscopic), and adjuvant chemotherapy (present or absent). Patients in the non-older and older groups were matched in a 1:1 ratio using the nearest propensity score on the logit scale. Statistical significance was set at *p* < 0.05. The JMP 16.1 statistical software for Macintosh (SAS Institute, Cary, NC, USA) was used to perform all analyses.

## Results

### Comparison of clinicopathological factors between the non-older and older groups

Table 1 presents the clinicopathological characteristics of the patients. Table 2 shows the association between age and clinicopathological factors. The mean age was 67.9 ± 8.49 years in the non-older group and 83.8 ± 3.40 years in the older group (*P* < 0.001). Older age (≥ 80 years) was significantly associated with female sex (*p* = 0.019), high ASA score (≥ 3, *p* < 0.001), lower preoperative serum albumin level (≤ 3.5 g/dL, *p* = 0.001), higher incidence of right-sided colon cancer (*p* = 0.005), poor differentiated histopathological type (*p* = 0.049), advanced pStage (stage II or III: *p* = 0.013), lymphatic invasion (*p* = 0.018), open surgery (*p* = 0.005), shorter operative time (*p* = 0.008), lower frequency of adjuvant chemotherapy (*p* < 0.001), and longer postoperative hospitalization (*p* = 0.041). The frequency of preoperative chemo-radio or chemotherapy and the proportions of elective and urgent surgery did not differ between the non-older and older groups. No significant difference was found in stoma creation, whereas the surgical procedure differed between the two groups. The incidence of severe postoperative complications did not differ between the two groups (*p* = 0.981). Details of the postoperative complications are presented in Additional file 1. No significant between-group differences were observed in the occurrence of any complications except for anastomotic leakage.

**Table 1:** Patient characteristics

Variables		Total patients (*n* = 760)	
**Age, years**		71.9	± 10.2
**Sex**	Female Male	331 429	(44%) (56%)
**BMI, kg/m** ^**2**^		22.2	± 3.56
**ASA-PS**	4 3 2 1	6 147 516 91	(1%) (19%) (68%) (12%)
**Treatment for colorectal obstruction**	Presence Absence	48 712	(6%) (94%)
**Location**	Right Left Rectum	273 236 251	(36%) (31%) (33%)
**Preoperative treatment** **(chemo-radio or chemo therapy)**	Presence Absence	11 749	(1%) (99%)
**Histopathological type** ^**a**^	por/sig/muc tub/pap	62 698	(8%) (92%)
**T stage** ^**b**^	T4 T3 T2 T1	138 377 115 130	(18%) (50%) (15%) (17%)
**N stage** ^**b**^	N2 N1 N0	85 199 476	(11%) (26%) (63%)
**pStage** ^**b**^	3 2 1	284 273 203	(37%) (36%) (27%)
**Lymphatic invasion**	Presence Absence	483 277	(64%) (36%)
**Venous invasion**	Presence Absence	420 340	(55%) (45%)
**Surgical approach**	Open Laparoscopic	226 534	(30%) (70%)
**Operative blood loss, ml**		139	± 281
**Operative time, min**		269	± 109
**Severe postoperative complication** ^**c**^	Presence Absence	56 704	(7%) (93%)
**Adjuvant chemotherapy**	Presence Absence	230 530	(30%) (70%)

a: According to the Japanese classification of colorectal carcinoma, 8th edition

b: According to the 7th edition of the UICC/TNM staging system

c: Grade 3 or higher according to the Clavien–Dindo classification

*tub* tubular adenocarcinoma, *pap* papillary adenocarcinoma, *por* poorly differentiated adenocarcinoma,

*sig* signet-ring cell carcinoma, *muc* mucinous adenocarcinoma, *ASA-PS* American Society of Anesthesiologists Physical Status, *BMI* body mass index

**Table 2:** Comparison of clinicopathological factors between non-older and older patients

		*n* = 760	Non-older group *n* = 569		Older group *n* = 191		*P*-value ^d^
**Age, years**			67.9	± 8.49	83.8	± 3.40	< 0.001
**Sex**	Female Male	331 429	234 335	(41%) (59%)	97 94	(51%) (49%)	0.019
**BMI, kg/m** ^**2**^	> 22 ≤ 22	389 371	302 267	(53%) (47%)	87 104	(46%) (54%)	0.071
**ASA-PS**	≥ 3 < 3	153 607	92 477	(16%) (84%)	61 130	(32%) (68%)	< 0.001
**Preoperative albumin level, g/dl**	≤ 3.5 > 3.5	159 601	103 466	(18%) (82%)	56 135	(29%) (71%)	0.001
**Location**	Right Left Rectum	273 236 251	186 187 196	(33%) (33%) (34%)	87 49 55	(45%) (26%) (29%)	0.005
**Preoperative treatment** **(chemo-radio or chemo therapy)**	Presence Absence	11 749	9 560	(2%) (98%)	2 189	(1%) (99%)	0.592
**Treatment for colorectal obstruction**	Presence Absence	48 712	33 536	(6%) (94%)	15 176	(8%) (92%)	0.312
**Histopathological type** ^**a**^	por/sig/muc tub/pap	62 698	40 529	(7%) (93%)	22 169	(12%) (88%)	0.049
**T stage** ^**b**^	T4 T1-3	138 622	107 462	(19%) (81%)	31 160	(16%) (84%)	0.424
**N stage** ^**b**^	N1-2 N0	284 476	215 354	(38%) (62%)	69 122	(36%) (64%)	0.681
**pStage** ^**b**^	II, III I	557 203	404 165	(71%) (29%)	153 38	(80%) (20%)	0.013
**Lymphatic invasion**	Presence Absence	483 277	348 221	(61%) (39%)	135 56	(71%) (29%)	0.018
**Venous invasion**	Presence Absence	420 340	309 260	(54%) (46%)	111 80	(58%) (42%)	0.359
**Surgical procedure**	Ileocecal resection Right hemicolectomy Transverse colectomy Left hemicolectomy Sigmoidectomy Hartmann operation Rectal resection	93 128 46 43 184 24 240	60 87 33 32 146 14 195	(11%) (15%) (6%) (6%) (26%) (2%) (34%)	33 41 13 11 38 10 45	(17%) (21%) (7%) (6%) (20%) (5%) (24%)	0.005
**Stoma**	Permanent stoma Diverting stoma Absence	69 22 669	48 18 503	(9%) (3%) (88%)	21 4 166	(11%) (2%) (87%)	0.442
**Elective or urgent surgery**	Elective Urgent	747 13	560 9	(98%) (2%)	187 4	(98%) (2%)	0.636
**Surgical approach**	Open Laparoscopic	226 534	154 415	(27%) (73%)	72 119	(38%) (62%)	0.005
**Operative blood loss, ml**	≥ 100 < 100	236 524	168 401	(30%) (70%)	68 123	(36%) (64%)	0.116
**Operative time, min**	≥ 250 < 250	377 383	298 271	(52%) (48%)	79 112	(41%) (59%)	0.008
**Severe postoperative complication** ^**c**^	Presence Absence	56 704	42 527	(8%) (92%)	14 177	(7%) (93%)	0.981
**Postoperative hospital stay, days**			16.2	± 12.2	18.6	± 18.3	0.041
**Adjuvant chemotherapy**	Presence Absence	230 530	207 362	(36%) (64%)	23 168	(12%) (88%)	< 0.001

a: According to the Japanese classification of colorectal carcinoma, 8th edition

b: According to the 7th edition of the UICC/TNM staging system

c: Grade 3 or higher according to the Clavien–Dindo classification

d: *P* values are from the chi-square test or Student’s t-test

*tub* tubular adenocarcinoma, *pap* papillary adenocarcinoma, *por* poorly differentiated adenocarcinoma, *sig* signet-ring cell carcinoma, *muc* mucinous adenocarcinoma, *ASA-PS* American Society of Anesthesiologists Physical Status, *BMI* body mass index

### Survival analysis

**Fig. 1:**
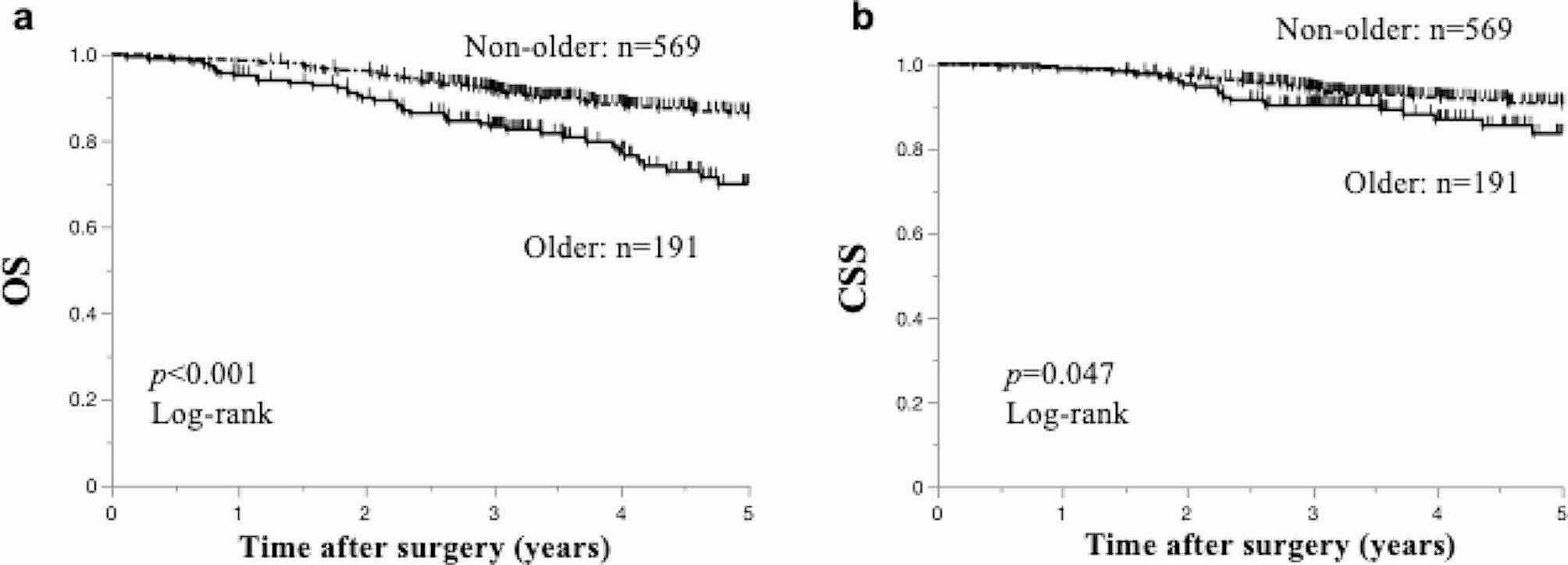
Association between prognosis and age in patients with CRC. Survival analysis according to age. Overall survival (**a**) and cancer-specific survival (**b**) using the Kaplan–Meier method are shown for non-older and older patients. CRC, colorectal cancer

The median postoperative follow-up duration was 49.4 and 40.8 months in the non-older and older patients, respectively. The older group showed significantly worse 5-year OS (69.8 vs. 85.9%; *p* < 0.001) and CSS (82.5% vs. 90.3%; *p* = 0.047) than the non-older group (Fig. 1).

Table 3 shows the associations between CSS and clinicopathological factors in 760 patients. The univariate and multivariate analyses revealed that high ASA score (≥ 3; hazard ratio [HR] = 2.47, 95% confidence interval [CI]: 1.45–4.20, *p* < 0.001), rectal cancer (HR = 2.10, 95% CI: 1.26–3.50, *p* = 0.004), deeper tumor invasion (pathological T4; HR = 3.19, 95% CI: 1.83–5.55, *p* < 0.001), and lymph node metastasis (pathological N1–2; HR = 2.99, 95% CI: 1.67–5.35, *p* < 0.001) were independent prognostic factors for CSS. In contrast, older age and postoperative complications were not prognostic factors for CSS.

**Table 3:** Univariate and multivariate analyses for cancer-specific survival

Variables		*n* = 760	Univariate			Multivariate		
			5-years CSS (%)	*P*-value ^d^		HR	95% CI	*P*-value ^e^
**Age, years**	≥ 80 < 80	191 569	82.5 90.3	0.047		1.65	0.95–2.87	0.070
**Sex**	Female Male	331 429	90.3 87.9	0.610				
**BMI, kg/m** ^**2**^	> 22 ≤ 22	389 371	90.0 87.6	0.444				
**ASA-PS**	≥ 3 < 3	153 607	75.6 91.4	< 0.001		2.47	1.45–4.20	< 0.001
**Location**	Rectum Colon	251 509	85.1 90.8	0.025		2.10	1.26–3.50	0.004
**Histopathological** **type** ^**a**^	por/sig/muc tub/pap	62 698	79.4 89.7	0.019		1.65	0.82–3.33	0.156
**T stage** ^**b**^	T4 T1-3	138 622	74.5 92.0	< 0.001		3.19	1.83–5.55	< 0.001
**N stage** ^**b**^	N1-2 N0	284 476	79.5 94.6	< 0.001		2.99	1.67–5.35	< 0.001
**Lymphatic invasion**	Presence Absence	483 277	85.7 94.2	0.001		1.40	0.70–2.79	0.331
**Venous invasion**	Presence Absence	420 340	86.2 92.0	0.049		1.15	0.64–2.06	0.630
**Severe postoperative complication** ^**c**^	Presence Absence	56 704	82.3 89.4	0.069				

a: According to the Japanese classification of colorectal carcinoma, 8th edition

b: According to the 7th edition of the UICC/TNM staging system

c: Grade 3 or higher according to the Clavien–Dindo classification

d: *P* values are from the log-rank test

e: *P* values are from Cox’s proportional hazard model

*CSS* Cancer-specific survival, *HR* Hazard ratio, *CI* Confidence interval, *tub* tubular adenocarcinoma, *pap* papillary adenocarcinoma, *por* poorly differentiated adenocarcinoma, *sig* signet-ring cell carcinoma, *muc* mucinous adenocarcinoma, *ASA-PS* American Society of Anesthesiologists Physical Status, *BMI* body mass index

### Prognostic impact of postoperative complications in elderly patients

**Fig. 2:**
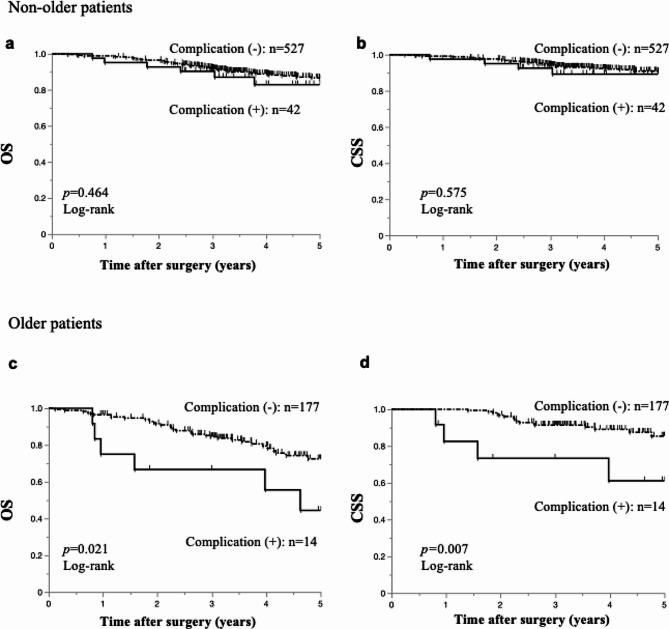
Association between prognosis and severe postoperative complication stratified based on age. **(a)** and **(b)** Survival analysis according to the severe postoperative complications that developed in non-older patients. Overall survival **(a)** and cancer-specific survival **(b)** using the Kaplan–Meier method are shown for non-older patients with or without severe postoperative complications. **(c)** and **(d)** Survival analysis according to the severe postoperative complications that developed in older patients. Overall survival **(c)** and cancer-specific survival **(d)** using the Kaplan–Meier method are shown for older patients with or without severe postoperative complications

Figure 2 shows the results of the survival analyses stratified based on age. The occurrence of complications did not affect the prognosis in the non-older group (Fig. 2a and b); in the older group, OS (*p* = 0.021; Fig. 2c) and CSS (*p* = 0.007; Fig. 2d) were significantly worse in patients with complications than in those without complications. Moreover, the correlation between the estimated cumulative recurrence rate and postoperative complications was analyzed (Additional file 2). The recurrence rates did not differ between patients with and without severe complications in the non-older group (17.6 vs. 25.6%; *p* = 0.371, Additional file 2a); meanwhile, the recurrence rates tended to be higher in patients with complications than in those without complications in the older group (17.4% vs. 35.1%; *p* = 0.113, Additional file 2b).

Table 4 shows the prognostic factors for CSS in older patients. The univariate and multivariate analyses revealed that deeper tumor invasion (pathological T4; HR = 4.12, 95% CI: 1.73–9.83, *p* = 0.001), lymph node metastasis (pathological N1–2; HR = 3.25, 95% CI: 1.25–8.40, *p* = 0.014), and postoperative complications (HR = 4.00, 95% CI: 1.27–12.6, *p* = 0.017) were independent prognostic factors for CSS. Older age (≥ 85 years) was not associated with poor prognosis in this analysis.

**Table 4:** Univariate and multivariate analyses for cancer-specific survival in older patients

Variables		*n* = 191	Univariate			Multivariate		
			5-years CSS (%)	*P*-value ^d^		HR	95% CI	*P*-value ^e^
**Age, years**	≥ 85 < 85	61 130	82.2 84.0	0.638				
**Sex**	Female Male	97 94	79.2 87.9	0.115				
**BMI, kg/m** ^**2**^	> 22 ≤ 22	87 104	87.4 80.1	0.318				
**ASA-PS**	≥ 3 < 3	61 130	69.6 88.3	0.156				
**Location**	Colon Rectum	136 55	83.7 83.8	0.939				
**Histopathological type** ^**a**^	por/sig/muc tub/pap	22 169	73.6 84.8	0.220				
**T stage** ^**b**^	T4 T1-3	31 160	57.2 88.8	< 0.001		4.12	1.73–9.83	0.001
**N stage** ^**b**^	N1-2 N0	69 122	73.7 89.5	0.002		3.25	1.25–8.40	0.014
**Lymphatic invasion**	Presence Absence	135 56	81.5 87.7	0.331				
**Venous invasion**	Presence Absence	111 80	78.8 89.4	0.299				
**Severe postoperative complication** ^**c**^	Presence Absence	14 177	61.1 85.3	0.007		4.00	1.27–12.6	0.017

a: According to the Japanese classification of colorectal carcinoma, 8th edition

b: According to the 7th edition of the UICC/TNM staging system

c: Grade 3 or higher according to the Clavien–Dindo classification

d: *P* values are from the log-rank test

e: *P* values are from Cox’s proportional hazard model

*CSS* Cancer-specific survival, *HR* Hazard ratio, *CI* Confidence interval, *tub* tubular adenocarcinoma, *pap* papillary adenocarcinoma, *por* poorly differentiated adenocarcinoma, *sig* signet-ring cell carcinoma, *muc* mucinous adenocarcinoma, *ASA-PS* American Society of Anesthesiologists Physical Status, *BMI* body mass index

### Propensity score matching analysis

To eliminate confounding factors for the occurrence of severe postoperative complications and prognosis, we performed both multivariate analysis and propensity score matching analysis. Additional file 3 shows clinicopathological factors after matching. The non-older and older groups were well-balanced with respect to covariates, except for age. In the matched cohort, the frequency of severe postoperative complication (*p* = 0.533, Additional file 3), OS (*p* = 0.063, Additional file 4a), and CSS (*p* = 0.384, Additional file 4b) did not significantly differ between non-older and older patients. Moreover, in the older group, OS (*p* = 0.039, Additional file 5c) and CSS (*p* = 0.033, Additional file 5d) were significantly worse in patients with complications than in those without complications, whereas the complications were not associated with prognosis in the non-older group (Additional file 5a and b).

## Discussion

Recent studies have demonstrated that laparoscopic resection of CRC in older patients is safe and feasible and can lead to favorable short- and long-term outcomes [5, 6, 15]. This study revealed that the incidence of severe postoperative complications did not differ between older and non-older patients with CRC. Although the prognosis of older patients was significantly worse than that of non-older patients, older age was not an independent prognostic factor for CSS.

Previous studies revealed that the occurrence of postoperative complications could be a prognostic factor for various malignancies, including CRC [7–9, 16, 17]. However, only a few studies have investigated the impact of complications on the prognosis of older patients compared with that of non-older patients. This study revealed that older patients with severe complications had significantly worse CSS and OS than those without complications; meanwhile, the occurrence of complications was not associated with prognosis in non-older patients. The multivariate analysis revealed that severe postoperative complications were independent risk factors for CSS in older patients with CRC.

Although this study demonstrated that older patients with postoperative complications showed poor prognosis, postoperative complications may have a negative physiological effect and shorten life expectancy [18]. Therefore, various factors may affect the prognosis of older patients. To exclude the influence of these factors, patients who experienced surgery-related mortality were excluded from this study, and the prognostic significance of severe complications was evaluated based on the CSS and cumulative recurrence rates. Moreover, the frequency of adjuvant chemotherapy was lower in older patients. Elderly patients sometimes choose not to receive adjuvant chemotherapy due to the higher risk of comorbidity, poor performance status, or insufficient social support [19, 20]. These factors may have contributed to the lower frequency of adjuvant chemotherapy in the present study. In our study, the use of adjuvant chemotherapy was not a prognostic predictor in older patients, and the frequency of adjuvant chemotherapy in older patients did not differ between patients with severe postoperative complications and those without severe postoperative complications (data not shown). Despite efforts to control for these factors, this study was not a prospective randomized trial, and these confounders were not completely eliminated.

It remains unclear why severe postoperative complications more strongly affect the prognosis of older patients than those of non-older patients. Postoperative complications induce systemic inflammation, which can cause host immunosuppression [21, 22]. Moreover, an excessive postoperative inflammatory response can promote cell growth or enhance the migratory or invasive abilities of residual cancer cells [23, 24]. These factors may contribute to immune escape by residual cancer cells and the occurrence of micrometastases, leading to postoperative recurrence. In contrast, previous studies demonstrated that older patients might experience enhanced postoperative inflammation and impaired immunonutritional status [25–29]. Consequently, the possible mechanisms of severe postoperative complications could strongly affect the prognosis of older patients.

In aging societies, the life expectancy of older individuals has relatively increased. For example, the life expectancies of 80-year-old women and men are 12.12 and 9.22 years, respectively, in Japan [30]. Therefore, radical surgery for CRC to achieve long-term survival is necessary not only for non-older patients but also for older patients. This study suggests that the oncological outcomes in older patients are improved by preventing the occurrence of severe postoperative complications. Previous studies have demonstrated that laparoscopic surgery is more beneficial in older patients with CRC than in younger patients [15, 31]. These findings suggest that minimally invasive surgeries, such as laparoscopic or robotic surgery, contribute to the improvement in the prognosis of older patients with CRC.

This study had certain limitations. First, considering the retrospective nature of this study, patients had CRC in various locations or stages. Therefore, several possible factors may affect the prognosis of patients with CRC. However, subgroup analysis based on the tumor location or surgical approach showed that these factors did not affect the association between age and postoperative complications. Moreover, for rectal cancer, neoadjuvant or postoperative adjuvant treatment were not associated with prognosis or the incidence of postoperative complication (data not shown). Second, a selection bias existed regarding the indications of the operative method, such as laparoscopic surgery, which changed during the recruitment period of this study. The proportion of patients who underwent laparoscopic surgery was higher in the late period than in the early period. Despite this difference, the study period was not associated with severe complications or prognosis (data not shown). Third, the sample size was relatively small to investigate its prognostic impact, especially in older patients with severe postoperative complications. Despite efforts to control for confounders using multivariate analysis and propensity score matching analysis, this was not a prospective randomized trial, and these confounders were not completely eliminated in this study. Therefore, further multicenter studies with large numbers of patients are needed to confirm our results.

## Conclusion

Colorectomy for CRC can be performed safely in both older and non-older patients. Severe postoperative complications may significantly affect the prognosis of older patients with CRC. Therefore, surgeons should manage various aspects of perioperative care to prevent the occurrence of severe postoperative complications and to prolong the life expectancy, even in older patients with CRC.

### Electronic supplementary material

Below is the link to the electronic supplementary material.

Supplementary Material 1

Supplementary Material 2

Supplementary Material 3

Supplementary Material 4

Supplementary Material 5

## Data Availability

The datasets generated and/or analyzed during the current study are not publicly available due to the personal information protection law in Japan but are available after the permission from the institutional review board and the corresponding author on reasonable request.

## Abbreviations

ASA: American Society of Anesthesiologists
CI: Confidence interval
CRC: Colorectal cancer
CSS: Cancer-specific survival
HR: Hazard ratio
JSCCR: Japanese Society for Cancer of the Colon and Rectum
OS: Overall survival
