# Supplementary material for: Impact of severe postoperative complications on the prognosis of older patients with colorectal cancer: a two-center retrospective study
Source: BMC Gastroenterol. 2024 Apr 2;24:125. doi: 10.1186/s12876-024-03213-y (PMC10988919; doi:10.1186/s12876-024-03213-y)
Supplement: Supplementary file 1 — Supplementary Material 1 [file 12876_2024_3213_MOESM1_ESM.docx]

**Additional file 1** Details of severe postoperative complications

| **Type of complication** | **Non elderly**  **n=569** | | **Elderly**  **n=191** | | **Total**  **n=760** |
| --- | --- | --- | --- | --- | --- |
| **Anastomotic leakage** | 21 | (4%) | 1 | (1%) | 0.028 |
| **Intra-abdominal abscess** | 6 | (1%) | 3 | (2%) | 0.898 |
| **Small bowel obstruction or ileus** | 5 | (1%) | 4 | (2%) | 0.239 |
| **Bleeding** | 4 | (1%) | 0 | (0%) | 0.577 |
| **Respiratory-related** | 1 | (1%) | 2 | (1%) | 0.157 |
| **Cardiovascular-related** | 1 | (1%) | 1 | (1%) | 0.439 |

File name: Additional file 1

File format: DOC

Title of data: Details of severe postoperative complications
