# Supplementary material for: Impact of severe postoperative complications on the prognosis of older patients with colorectal cancer: a two-center retrospective study
Source: BMC Gastroenterol. 2024 Apr 2;24:125. doi: 10.1186/s12876-024-03213-y (PMC10988919; doi:10.1186/s12876-024-03213-y)
Supplement: Supplementary file 2 — Supplementary Material 2 [file 12876_2024_3213_MOESM2_ESM.pptx]

## Slide 1
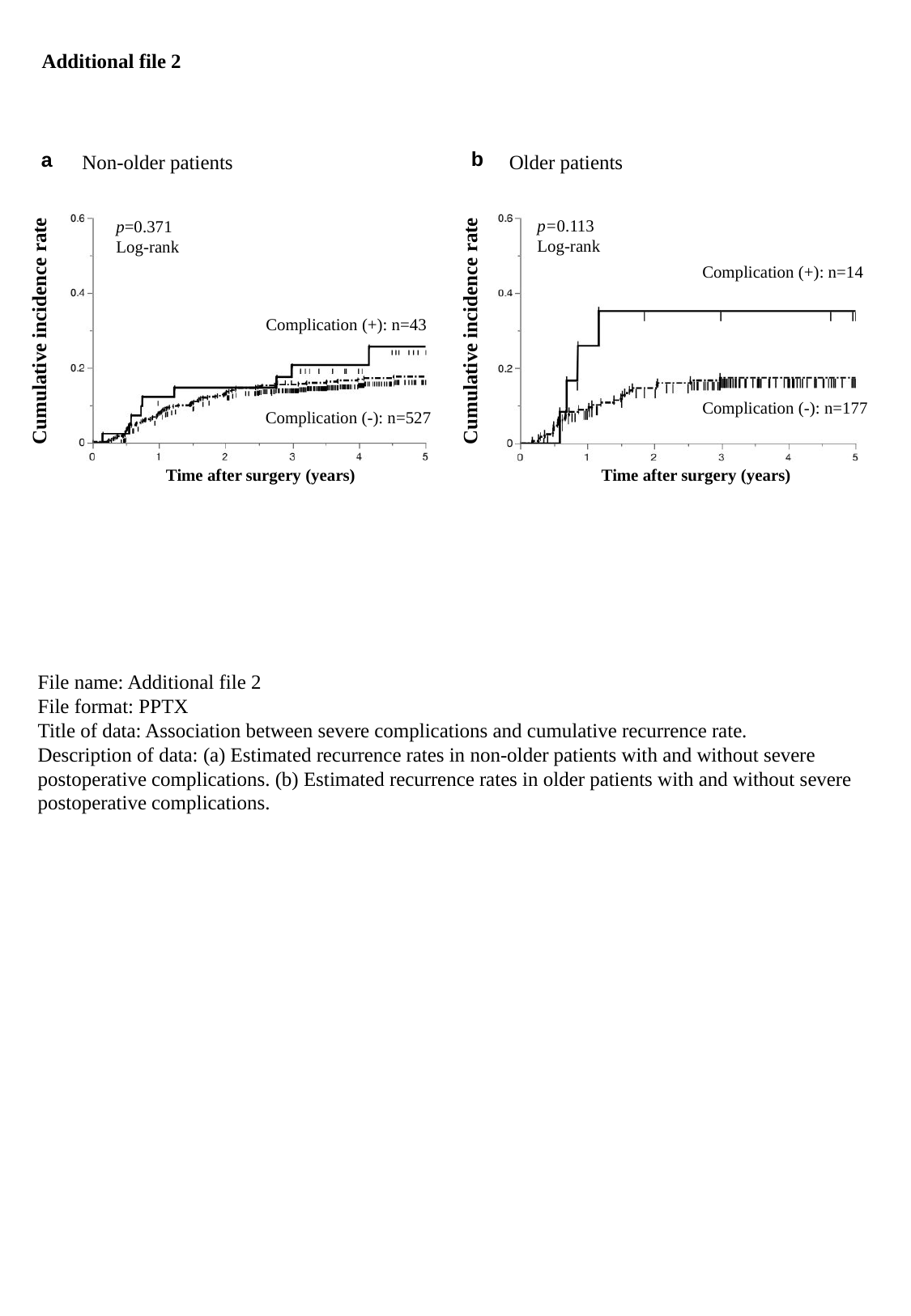

Additional file 2
b
a
Non-older patients
Older patients
Cumulative incidence rate
Cumulative incidence rate
p=0.113
Log-rank
p=0.371
Log-rank
Complication (+): n=14
Complication (+): n=43
Complication (-): n=177
Complication (-): n=527
Time after surgery (years)
Time after surgery (years)
File name: Additional file 2
File format: PPTX
Title of data: Association between severe complications and cumulative recurrence rate.
Description of data: (a) Estimated recurrence rates in non-older patients with and without severe postoperative complications. (b) Estimated recurrence rates in older patients with and without severe postoperative complications.
