# Supplementary material for: Impact of severe postoperative complications on the prognosis of older patients with colorectal cancer: a two-center retrospective study
Source: BMC Gastroenterol. 2024 Apr 2;24:125. doi: 10.1186/s12876-024-03213-y (PMC10988919; doi:10.1186/s12876-024-03213-y)
Supplement: Supplementary file 3 — Supplementary Material 3 [file 12876_2024_3213_MOESM3_ESM.docx]

**Additional file 3** Comparison of clinicopathological factors between non-older and older patients after matching

|  | | **n=348** | **Non-older group**  **n=174** | | **Older group**  **n=174** | | ***P*-value ^d^** |
| --- | --- | --- | --- | --- | --- | --- | --- |
| **Age, years** |  |  | 68.7 | ±8.57 | 83.6 | ±3.35 | < 0.001 |
| **Sex** | Female  Male | 175  173 | 89  85 | (51%)  (49%) | 86  88 | (49%)  (51%) | 0.747 |
| **BMI, kg/m^2^** | > 22  ≤ 22 | 155  193 | 74  100 | (43%)  (57%) | 81  93 | (47%)  (53%) | 0.450 |
| **ASA-PS** | ≥ 3  < 3 | 95  253 | 46  128 | (26%)  (74%) | 49  125 | (28%)  (72%) | 0.718 |
| **Location** | Right  Left  Rectum | 143  95  110 | 70  47  57 | (40%)  (27%)  (33%) | 73  48  53 | (42%)  (28%)  (30%) | 0.896 |
| **Preoperative treatment**  **(chemo-radio or chemo therapy)** | Presence  Absence | 8  340 | 6  168 | (3%)  (97%) | 2  172 | (1%)  (99%) | 0.152 |
| **Treatment for colorectal obstruction** | Presence  Absence | 27  321 | 14  160 | (8%)  (92%) | 13  161 | (7%)  (93%) | 0.841 |
| **Histopathological type ^a^** | por / sig / muc  tub / pap | 25  323 | 9  165 | (5%)  (95%) | 16  158 | (9%)  (91%) | 0.146 |
| **T stage ^b^** | T4  T1-3 | 50  298 | 24  150 | (14%)  (86%) | 26  148 | (15%)  (85%) | 0.759 |
| **N stage ^b^** | N1-2  N0 | 113  235 | 56  118 | (32%)  (68%) | 57  117 | (33%)  (67%) | 0.908 |
| **pStage ^b^** | II, III  I | 261  87 | 123  51 | (71%)  (29%) | 138  36 | (79%)  (21%) | 0.063 |
| **Lymphatic invasion** | Presence  Absence | 226  122 | 105  69 | (60%)  (40%) | 121  53 | (70%)  (30%) | 0.072 |
| **Venous invasion** | Presence  Absence | 186  162 | 86  88 | (49%)  (51%) | 100  74 | (57%)  (43%) | 0.132 |
| **Surgical procedure** | Ileocecal resection  Right hemicolectomy  Transverse colectomy  Left hemicolectomy  Sigmoidectomy  Hartmann operation  Rectal resection | 54  62  25  19  74  13  101 | 26  29  13  8  37  4  57 | (15%)  (16%)  (7%)  (5%)  (21%)  (2%)  (33%) | 28  33  12  11  37  9  44 | (16%)  (19%)  (7%)  (7%)  (21%)  (5%)  (25%) | 0.617 |
| **Stoma** | Permanent stoma  Diverting stoma  Absence | 31  8  309 | 14  4  156 | (8%)  (2%)  (90%) | 17  4  153 | (10%)  (2%)  (88%) | 0.852 |
| **Elective or urgent surgery** | Elective  Urgent | 345  3 | 173  1 | (99%)  (1%) | 172  2 | (99%)  (1%) | 0.562 |
| **Surgical approach** | Open  Laparoscopic | 110  238 | 51  123 | (29%)  (71%) | 59  115 | (34%)  (66%) | 0.356 |
| **Operative blood loss, ml** | ≥ 100  < 100 | 104  244 | 46  128 | (26%)  (74%) | 58  116 | (33%)  (67%) | 0.159 |
| **Operative time, min** | ≥ 250  < 250 | 161  187 | 87  87 | (50%)  (50%) | 74  100 | (43%)  (57%) | 0.162 |
| **Severe postoperative complication ^c^** | Presence  Absence | 25  323 | 14  160 | (8%)  (92%) | 11  163 | (6%)  (94%) | 0.533 |
| **Postoperative hospital stay, days** |  |  | 16.8 | ±13.7 | 18.5 | ±18.7 | 0.350 |
| **Adjuvant chemotherapy** | Presence  Absence | 42  306 | 19  155 | (11%)  (89%) | 23  151 | (13%)  (87%) | 0.510 |

a: According to the Japanese classification of colorectal carcinoma 8th Edition

b: According to the 7th edition of UICC/TNM staging system

c: Grade 3 or higher according to the Clavien-Dindo classification

d: *P* values are from Chi-squared test or Student’s t-test.

*tub* tubular adenocarcinoma, *pap* papillary adenocarcinoma*, por* poorly differentiated adenocarcinoma, *sig* signet-ring cell carcinoma*, muc* mucinous adenocarcinoma

File name: Additional file 3

File format: DOC

Title of data: Comparison of clinicopathological factors between non-older and older patients after matching
