# Supplementary material for: Impact of severe postoperative complications on the prognosis of older patients with colorectal cancer: a two-center retrospective study
Source: BMC Gastroenterol. 2024 Apr 2;24:125. doi: 10.1186/s12876-024-03213-y (PMC10988919; doi:10.1186/s12876-024-03213-y)
Supplement: Supplementary file 4 — Supplementary Material 4 [file 12876_2024_3213_MOESM4_ESM.pptx]

## Slide 1
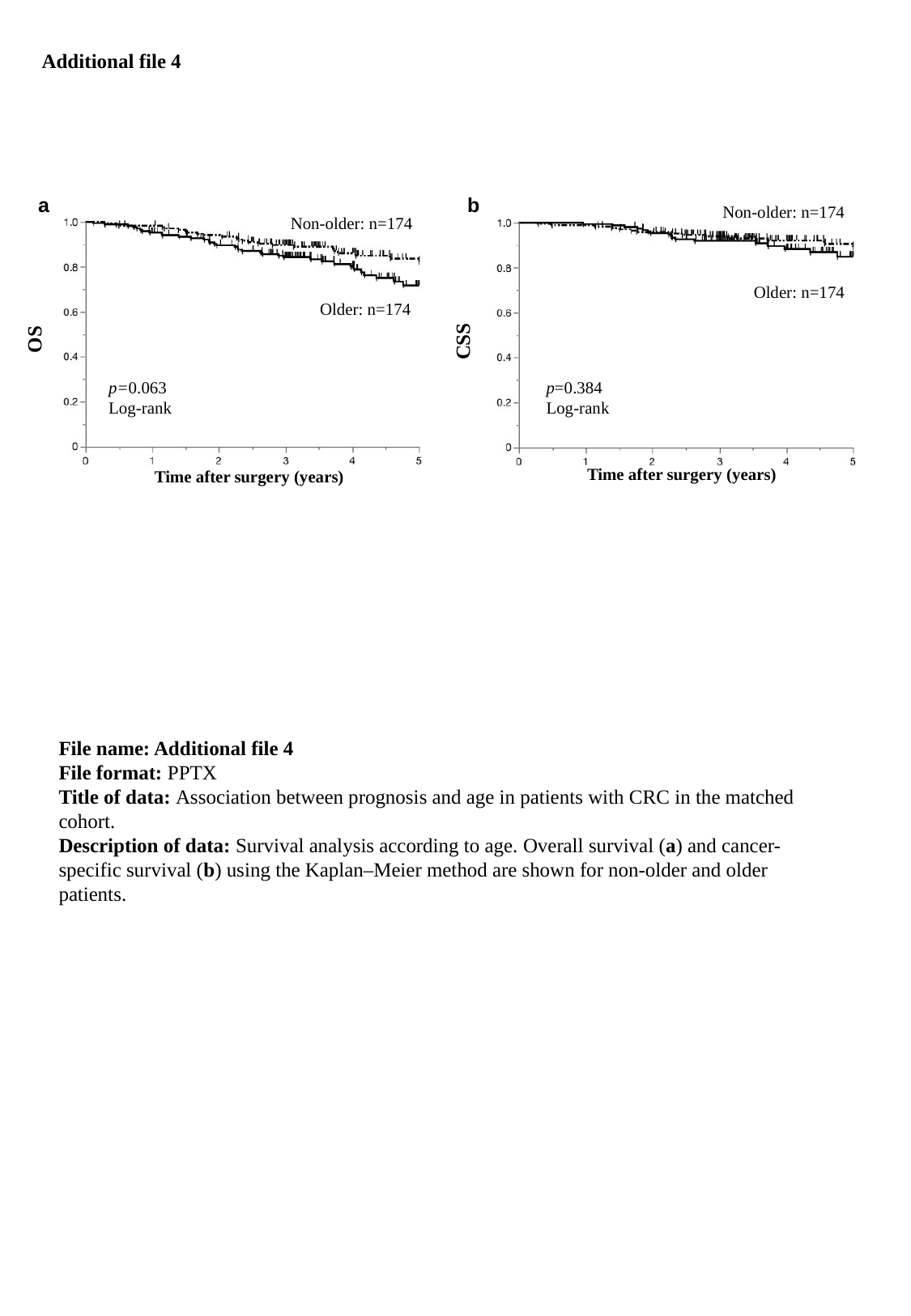

Additional file 4
b
a
Non-older: n=174
Non-older: n=174
CSS
Older: n=174
Older: n=174
OS
p=0.384
Log-rank
p=0.063
Log-rank
Time after surgery (years)
Time after surgery (years)
File name: Additional file 4
File format: PPTX
Title of data: Association between prognosis and age in patients with CRC in the matched cohort.
Description of data: Survival analysis according to age. Overall survival (a) and cancer-specific survival (b) using the Kaplan–Meier method are shown for non-older and older patients.
