# Supplementary material for: Impact of severe postoperative complications on the prognosis of older patients with colorectal cancer: a two-center retrospective study
Source: BMC Gastroenterol. 2024 Apr 2;24:125. doi: 10.1186/s12876-024-03213-y (PMC10988919; doi:10.1186/s12876-024-03213-y)
Supplement: Supplementary file 5 — Supplementary Material 5 [file 12876_2024_3213_MOESM5_ESM.pptx]

## Slide 1
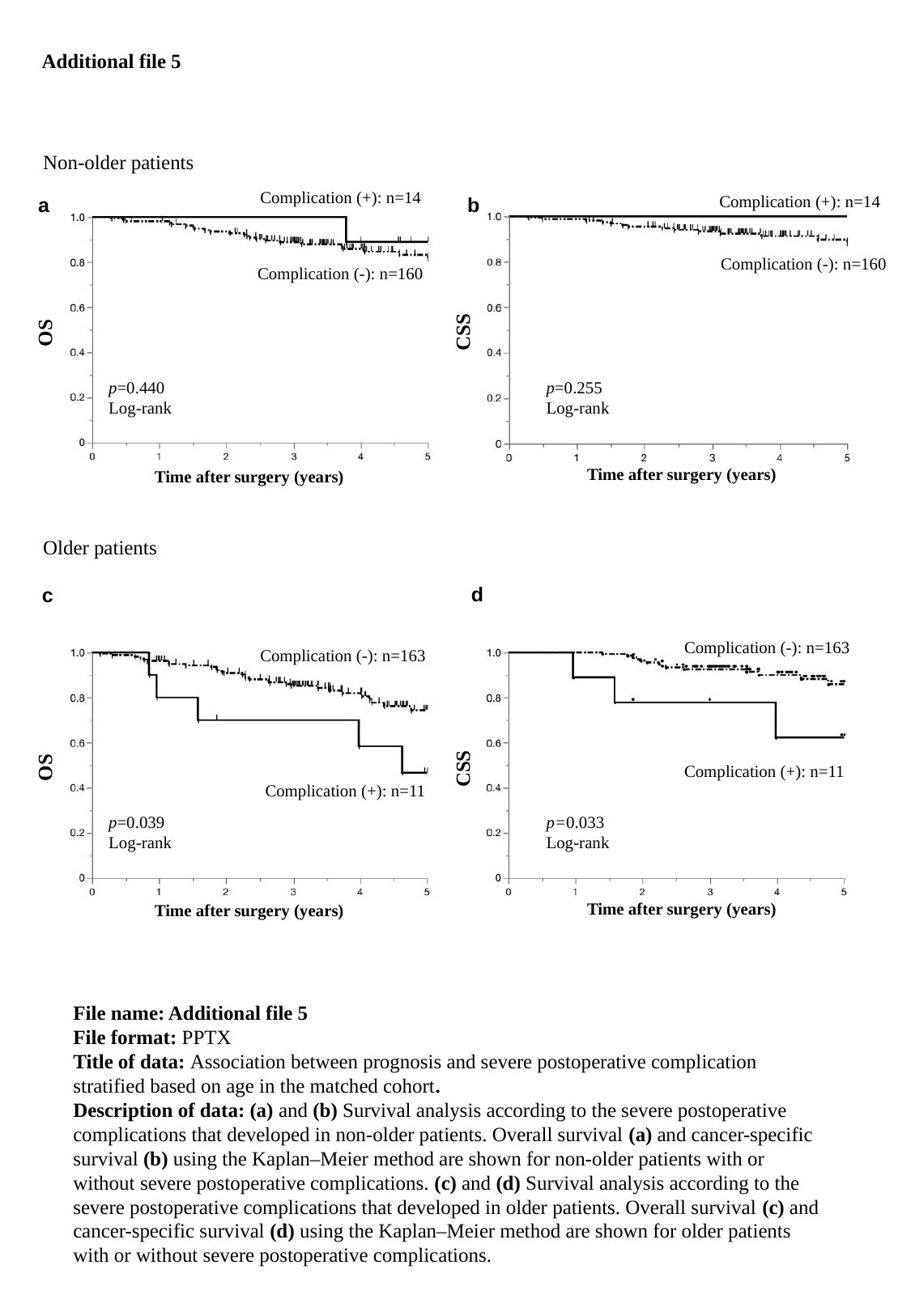

Additional file 5
Non-older patients
Complication (+): n=14
Complication (+): n=14
b
a
Complication (-): n=160
CSS
Complication (-): n=160
OS
p=0.440
Log-rank
p=0.255
Log-rank
Time after surgery (years)
Time after surgery (years)
Older patients
d
c
Complication (-): n=163
Complication (-): n=163
CSS
OS
Complication (+): n=11
Complication (+): n=11
p=0.033
Log-rank
p=0.039
Log-rank
Time after surgery (years)
Time after surgery (years)
File name: Additional file 5
File format: PPTX
Title of data: Association between prognosis and severe postoperative complication stratified based on age in the matched cohort.
Description of data: (a) and (b) Survival analysis according to the severe postoperative complications that developed in non-older patients. Overall survival (a) and cancer-specific survival (b) using the Kaplan–Meier method are shown for non-older patients with or without severe postoperative complications. (c) and (d) Survival analysis according to the severe postoperative complications that developed in older patients. Overall survival (c) and cancer-specific survival (d) using the Kaplan–Meier method are shown for older patients with or without severe postoperative complications.
